# Examining geographical inequalities for malaria outcomes and spending on malaria in 40 malaria-endemic countries, 2010–2020

**DOI:** 10.1186/s12936-024-05028-4

**Published:** 2024-07-10

**Authors:** Angela E. Apeagyei, Nishali K. Patel, Ian Cogswell, Kevin O’Rourke, Golsum Tsakalos, Joseph Dieleman

**Affiliations:** Institute for Health Metrics and Evaluation, 3980 15th Ave NE, Seattle, WA 98195 USA

**Keywords:** Inequality, Malaria, Development assistance for health, Health systems, Resource allocation

## Abstract

**Background:**

While substantial gains have been made in the fight against malaria over the past 20 years, malaria morbidity and mortality are marked by inequality. The equitable elimination of malaria within countries will be determined in part by greater spending on malaria interventions, and how those investments are allocated. This study aims to identify potential drivers of malaria outcome inequality and to demonstrate how spending through different mechanisms might lead to greater health equity.

**Methods:**

Using the Gini index, subnational estimates of malaria incidence and mortality rates from 2010 to 2020 were used to quantify the degree of inequality in malaria burden within countries with incidence rates above 5000 cases per 100,000 people in 2020. Estimates of Gini indices represent within-country distributions of disease burden, with high values corresponding to inequitable distributions of malaria burden within a country. Time series analyses were used to quantify associations of malaria inequality with malaria spending, controlling for country socioeconomic and population characteristics.

**Results:**

Between 2010 and 2020, varying levels of inequality in malaria burden within malaria-endemic countries was found. In 2020, values of the Gini index ranged from 0.06 to 0.73 for incidence, 0.07 to 0.73 for mortality, and 0.00 to 0.36 for case fatality. Greater total malaria spending, spending on health systems strengthening for malaria, healthcare access and quality, and national malaria incidence were associated with reductions in malaria outcomes inequality within countries. In addition, government expenditure on malaria, aggregated government and donor spending on treatment, and maternal educational attainment were also associated with changes in malaria outcome inequality among countries with the greatest malaria burden.

**Conclusions:**

The findings from this study suggest that prioritizing health systems strengthening in malaria spending and malaria spending in general especially from governments will help to reduce inequality of the malaria burden within countries. Given heterogeneity in outcomes in countries currently fighting to control malaria, and the challenges in increasing both domestic and international funding allocated to control and eliminate malaria, the efficient targeting of limited resources is critical to attain global malaria eradication goals.

**Supplementary Information:**

The online version contains supplementary material available at 10.1186/s12936-024-05028-4.

## Background

Substantial gains have been made in the fight against malaria in the past 20 years. According to the Global Burden of Disease study, between 2000 and 2021 the global incidence of malaria fell by an estimated 21.1%, while malaria mortality decreased by 32.6% [1]. These gains have been made in large part because of increased coverage of malaria treatment, insecticide-treated nets (ITN), indoor residual spraying (IRS), and chemoprevention, notably in sub-Saharan Africa, where malaria transmission remains the most pronounced [2]. Despite this, recent progress towards global malaria elimination has largely stalled due to competing domestic health priorities [3], plateauing donor funding [4], disruption in control and treatment activities due to the COVID-19 pandemic [5, 6] and inequities in burden, coverage, and utilization of services particularly in hard-to-reach populations and at the geographical margins [5, 7–9].

Global health initiatives increasingly emphasize health equity as a key determinant to achieving universal health coverage. In the case of malaria, the World Health Assembly endorsed the Global Technical Strategy for Malaria, which advocates for the inclusion of malaria interventions as part of universal health care and acknowledges the importance of adapting the malaria response to suit the context in each country [10]. Geographic gradients and progress towards equity in malaria control have been documented in several broad strands of the literature. For example, multiple studies using population-based surveys have demonstrated disparities in malaria transmission, coverage and outcomes, where outcomes capture the result of interventions, such as incidence and case fatality rates, across socioeconomic status [11–13]. While the drivers of inequality in malaria outcomes are routinely examined as part of national control programs, less frequently evaluated are the drivers of geographical differences in treatment outcomes among endemic countries, with available evidence mostly comprising of national or regional assessments of spatial variation [14–17].

While domestic and international financing for malaria rose considerably between 2000 and 2016 [18, 19], investments in malaria still fall short of targets set by the World Health Organization. The overall funding gap has increased from $2.6 billion in 2019 to $3.5 billion in 2020 and $3.8 billion in 2021 [20]. Achieving progress in the equity of malaria outcomes between and within countries requires efficient use of investments and the optimal allocation of resources in malaria control and elimination programmes [19, 21, 22]. To date, however, the association between malaria spending and the inequitable distribution of malaria outcomes within countries has not been comprehensively assessed.

In this study, state-level estimates among 40 malaria-endemic countries are used to demonstrate the magnitude and drivers of inequalities in malaria. Additionally, the relationship between inequality and malaria spending from domestic and foreign sources, disaggregated malaria spending by functional area, maternal education, health systems performance, economic status, and infectious disease risk measured at the country level are assessed. The study findings highlight associations between malaria outcome inequality and spending on malaria and demonstrate how spending through different mechanisms might lead to greater health equity.

## Methods

This analysis was conducted in 40 malaria-endemic countries with the greatest incidence rates defined in this study as countries with incidence rates greater than 5000 cases per 100,000 in 2020 (see Table 1). The analysis focuses on this subset of endemic countries, as geographic disparities could indicate limited access to necessary prevention and treatment to malaria at the state level. The study focuses on high endemic countries and exclude low endemic countries because countries with little malaria incidence tend to have high GINI values not because of great inequality per se but largely because their malaria incidence is low in general within the country.

**Table 1:** Malaria-endemic countries included in this analysis

Latin America and Caribbean	North Africa and Middle East	Southeast Asia, East Asia, and Oceania
Guyana	Sudan	Papua New Guinea
	Yemen	Solomon Islands^a^

**Sub-Saharan Africa **		
Angola^a^	Gabon^a^	Niger^a^
Benin^a^	The Gambia	Nigeria^a^
Burkina Faso^a^	Ghana	Senegal
Burundi^a^	Guinea^a^	Sierra Leone^a^
Cameroon^a^	Guinea-Bissau	Somalia
Central African Republic^a^	Kenya	South Sudan^a^
Chad	Liberia^a^	Togo^a^
Congo	Madagascar	Uganda^a^
Côte d'Ivoire^a^	Malawi	United Republic of Tanzania
Democratic Republic of the Congo^a^	Mali^a^	Zambia
Djibouti	Mauritania	Zimbabwe
Equatorial Guinea	Mozambique^a^	

^a^Malaria-endemic countries included in the stratified analysis

### Definition of geographic disparities

Data on total malaria cases (combined *Plasmodium falciparum* and *Plasmodium vivax*) and deaths (*P. falciparum*) at the first administrative level (admin1) from 2010 to 2020 were obtained from the Malaria Atlas Project (MAP) (www.map.ox.ac.uk). Using these data, malaria incidence, mortality, and case fatality for each first administrative unit were calculated. To estimate incidence and mortality rates, the number of cases or deaths (numerator) was divided by the population (denominator). To estimate (*P. falciparum*) case fatality ratios, mortality rates were divided by incidence rates to provide a population-based indicator of survival. These values were then used to compute the outcome measures of interest for this study.

For our outcome measures—incidence and case fatality—the *Gini coefficient* (also known as the Gini index) derived from the Lorenz curve [23] was adapted as a proxy for the relative difference in malaria incidence or case fatality within each malaria-control country (Appendix Section B). Although the Gini coefficient is most commonly used in studies on wealth and income inequality, it has been employed to analyse health disparities, including disease burden [15, 17, 24], life expectancy [25], inequality in particulate matter 2.5-related health outcomes [26], and DTP3 vaccine coverage [27]. The Gini coefficient is defined as:

$$G = 2\left( { \mathop \sum \limits_{i = 1}^{N} \frac{1}{2}\left( {P_{i} - P_{i - 1} } \right)\left( {C_{i} + C_{i - 1} } \right) } \right) - 1,$$

where $G$ is the Gini coefficient, $i$ is the admin1 unit of interest, $N$ is the number of admin1 units in a given country, $P_{i}$ is the cumulative proportion of the population in the $i{\text{th}}$ unit, and $C_{i}$ is the cumulative proportion of malaria outcomes in unit $i{\text{th}}$ unit. For each of the outcomes (incidence and case fatality), the Gini coefficient was calculated for each country and year by first ranking subnational units in decreasing order of the variable. The values of the Gini coefficients range from 0 in complete equality to 1 in complete inequality. When applied to malaria incidence, a Gini coefficient of 0 represents perfectly equitable distribution of malaria incident cases within countries and a Gini coefficient of 1 is interpreted as all malaria incident cases concentrated in one subnational unit. When applied to malaria case fatality, a Gini coefficient of 0 indicates that deaths due to malaria are equal across subnational units, whereas a coefficient of 1 indicates that deaths due to malaria are concentrated in one subnational unit.

### Malaria spending

The main predictor variables are total, government, and disaggregated malaria spending (i.e., spending broken down by program type). Data on total, government, and disaggregated malaria spending from 2010 to 2020 were extracted from the Institute for Health Metrics and Evaluation (IHME)’s Financing Global Health databases [28–31].

Total malaria spending consisted of government health expenditure (GHE), out-of-pocket (OOP) expenditure, prepaid private (PPP) expenditure, and development assistance for health (DAH) for malaria. Total malaria spending data were real currency converted to 2021 USD and expressed as spending per person in the population-at-risk. Spending per person at risk population was defined as total malaria spending divided by the World Malaria Report estimates of population at risk. The World Malaria Report estimates of population at risk is calculated using proportion of population at high, low, and no risk of malaria transmission provided by country-level national malaria control programmes.

Government malaria expenditure as a proportion of total malaria spending was included as a covariate, to provide an indication of a country’s social and political decisions as to how much funding should be allocated to malaria relative to the overall health budget.

Government expenditure on malaria and DAH for malaria were disaggregated by programme type [18, 31], including insecticide-treated nets (ITNs), indoor residual spraying (IRS), anti-malarial medicines, drug resistance, other vector control, human resources and technical assistance (HRTA), procurement and supply management (PSM), planning, administration, and overheads (PAO), infrastructure and equipment (IE), monitoring and evaluation (ME), other health systems strengthening, and other malaria programmes.

For the purpose of this analysis, disaggregated spending categories were combined to create three variables that captured spending on prevention, treatment, and health systems strengthening (Table 2). Programmes from which spending was categorized as prevention included ITNs, IRS, and other types of vector control. Programmes from which spending was categorized as treatment included anti-malarial medicines and drug resistance. Programmes from which spending was categorized as health systems strengthening (HSS) included HRTA, PSM, PAO, IE, ME, and other health systems strengthening. The three disaggregated spending variables were expressed as proportions of aggregated government expenditure on malaria and DAH for malaria.

**Table 2:** List of covariates used for analysis

Covariate	Indicator	Source
Total malaria spending^a^	Total malaria spending per person in the population-at-risk (2021 USD)	IHME [31]
Government expenditure on malaria	Government expenditure on malaria as a proportion of total malaria spending	IHME [31]
Spending on malaria prevention	Spending on ITNs, IRS, and other vector control as a proportion of government expenditure on malaria and DAH for malaria	IHME [31]
Spending on malaria treatment	Spending on antimalarials and drug resistance as a proportion of government expenditure on malaria and DAH for malaria	IHME [31]
Spending on health systems strengthening for malaria	Spending on HRTA, PSM, PAO, IE, ME, and other HSS as a proportion of government expenditure on malaria and DAH for malaria	IHME [31]
GDP per person^a^	GDP/population (2021 USD)	IHME [36]
Working age population	Age dependency ratio (as a % of working age population)	World Bank [37]
Health systems performance	Healthcare Access and Quality Index	IHME [38]
Socioeconomic status	Average years of maternal education	IHME [38]
Infectious disease risk	Population-weighted mean temperature	IHME [38]
Risk of malaria	National malaria incidence rate	[1]

^a^Total malaria spending and GDP are modelled as log-transformed values

### Other drivers of geographic disparities

The study analyses controlled for other potential socioeconomic and demographic drivers of geographic disparities ($X_{it}$) in malaria outcomes (Table 2). Covariates include the gross domestic product (GDP) per person, age dependency ratio, the Healthcare Access and Quality (HAQ) Index, average maternal education, population-weighted mean temperature, and national malaria incidence (Table 2). The covariates included were primarily informed by a conceptual framework from the WHO’s commission on the Social Determinants of Health (SDoH). This framework captures the primary structural and socioeconomic inputs that drive equity in health and wellbeing. Additional covariates were also included from the literature based on their established importance with health outcomes and the outputs of interest [32–38].

### Statistical analyses

Overall, malaria spending and GDP per person were log-transformed. These two variables were log-transformed to normalize the distribution and to allow for plausible interpretation of their respective coefficients [39]. From 2010 to 2020, within-country disparities in malaria over time are found and and the trends in disparities over time plotted. Furthermore, a panel dataset for 40 malaria-endemic countries from 2010 to 2020 is constructed. The following equation was used to estimate overall geographic inequality in malaria outcomes in order to quantify associations with changes in within-country geographic disparities.

$$\begin{aligned} g_{it} & = \alpha_{i} + \gamma_{t} + \beta_{0} + \beta_{1} TotalMalSpend_{it} + \beta_{2} \frac{{GovMalSpend_{it} }}{{TotalMalSpend_{it} }} \\ & \quad + \beta \frac{{DisagMalSpend_{it} }}{{GovMalSpend_{it} + DAHMalSpend_{it} }} + \beta X_{it} + \varepsilon_{it} , \\ \end{aligned}$$

where the Gini index, $g_{it}$, in country $i$ at year $t$ is a function of total spending on malaria ($TotalMalSpend$). Using this equation, when the independent variable is log transformed $\beta_{1}$—the coefficient of one of our variables of interest—can be interpreted as a 10% increase in total malaria spending is associated with a $\beta_{1} \times {\text{log}}\left( {1.10} \right)$ unit change in the dependent ($g_{it}$) variable. For government and disaggregated malaria spending, the other variables of interest, the coefficient can be interpreted as a 10% increase in the independent variable is associated with a $\beta \times 10^{2}$ percent change in the dependent variable. The standard errors of the coefficients were adjusted for using Huber-White robust adjustment to control for heteroscedasticity across panels and are clustered by years to address any serial correlation over time. Fixed effects on country ($\alpha_{i}$) and year ($\gamma_{t}$) were used to account for any unobserved confounding factors which vary by country and over time. It is important to note that these evaluations are all descriptive and not causal. All analyses were completed using R (version 4.2.1).

## Results

**Fig. 1:**
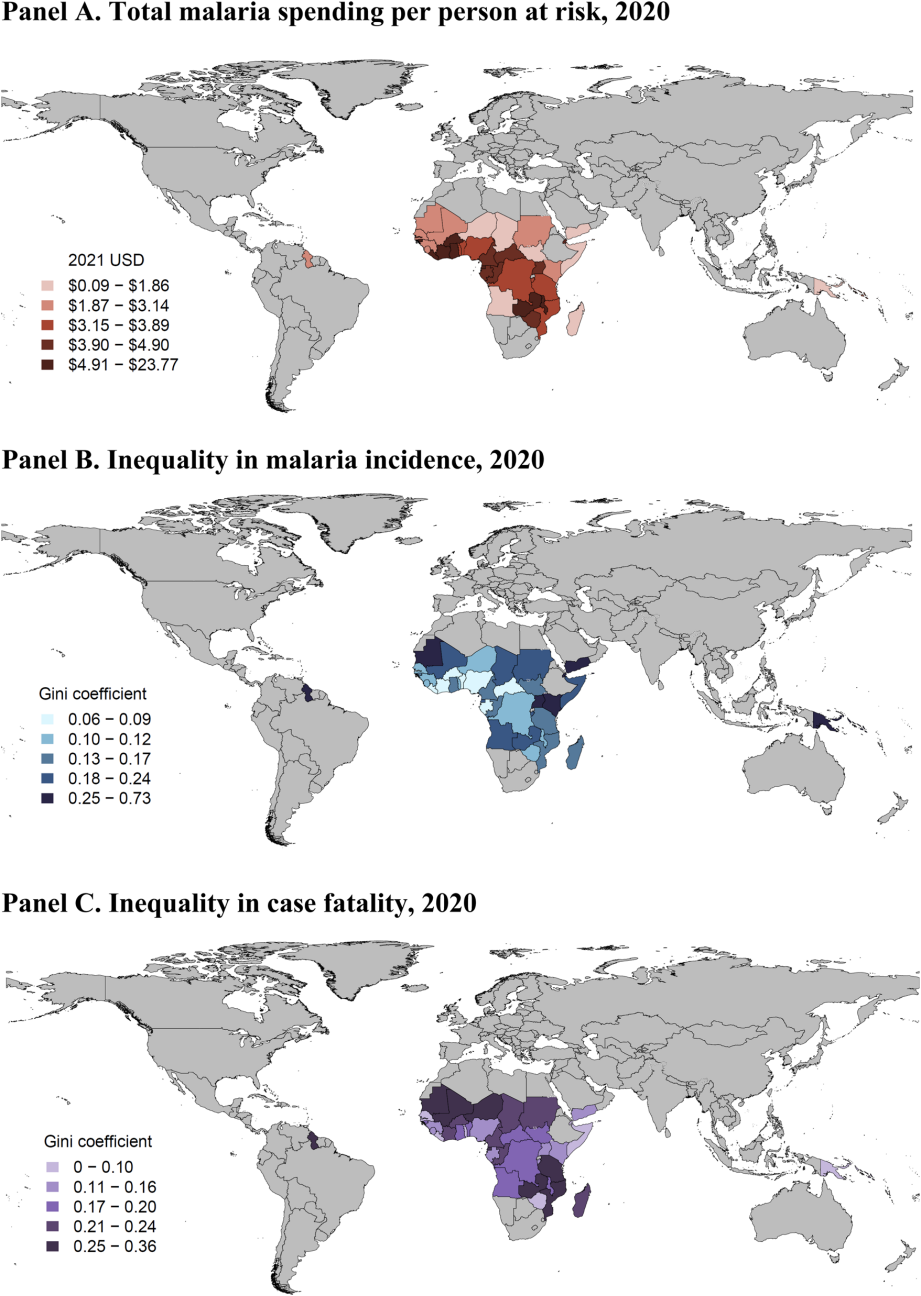
Comparison between total malaria spending (**A**) and inequality in incidence (**B**) and case fatality (**C**), 2020

The study found variation in geographic disparities in malaria incidence and case fatality across malaria-endemic countries (Fig. 1). In 2020, values for within-country Gini coefficient ranged from 0.06 to 0.73 for incidence and 0.00 to 0.36 for case fatality. Liberia, Burkina Faso, and Benin had the least incidence inequality (< 0.076), while Djibouti, Yemen, and Guyana had Gini coefficients greater than 0.42 (Fig. 1B). Inequality appears to be less pronounced when case fatality is considered. For case fatality, Djibouti, The Gambia, and Papua New Guinea had the least inequality (< 0.0085) whereas Mauritania, Guyana, and Mali had coefficient values greater than 0.32 (Fig. 1C). Countries with the lowest total malaria spending per person at risk—including Somalia, Yemen, Madagascar, and South Sudan—had consistently high incidence inequality levels (Fig. 1A).

**Fig. 2:**
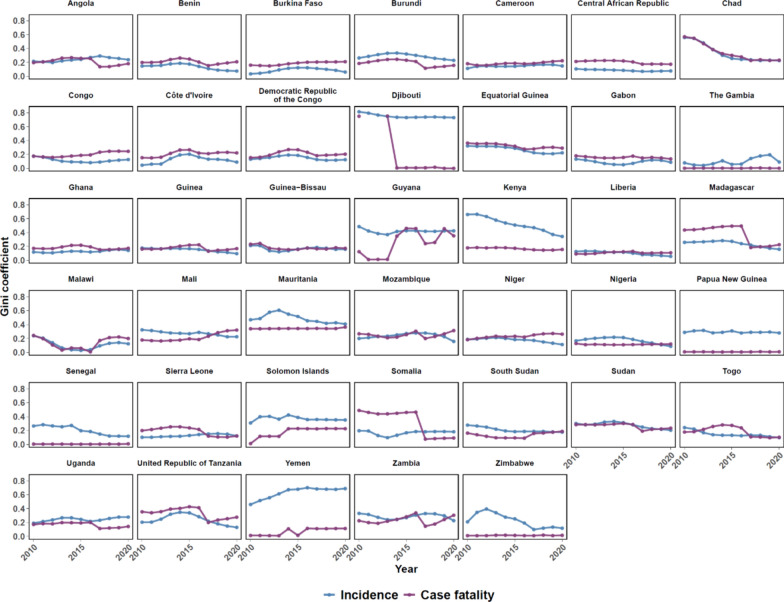
Country-specific trends in inequality, 2010–2020. There were no deaths reported in all Djibouti subnational units in 2011 and 2012

Country inequality time trends between 2010 and 2020 are illustrated in Fig. 2. Overall, country inequality decreased during the study period on average. The Gini coefficient peaks at 0.81 for incidence and 0.75 for case fatality throughout the time series. Across the two outcomes, countries have varying baseline values with greater variation in values for the Gini coefficient for case fatality than for incidence. Some countries also experience steep gradients at specific time points such as Djibouti, Guyana, Madagascar, and Somalia while other countries maintained relatively constant coefficients through the entire time spectrum including Cameroon, Central African Republic, Liberia, and Papua New Guinea.

Table 3 summarizes the analysis exploring potential determinants of inequality using models to assess changes within a country, over time, controlling for all time-invariant country characteristics. The study found significant associations for total malaria spending, proportion sourced by government, proportion spent on health systems strengthening, national malaria incidence, Healthcare Access and Quality Index, maternal education, and population-weighted mean temperature.

**Table 3:** Regression results using within effects (admin1 state-level outcomes)

	Incidence inequality	Case fatality inequality
ln(total malaria spending)	− 0.024 (0.008)**	− 0.033 (0.013)**
Government malaria expenditure	− 0.045 (0.029)	0.198 (0.048)***
Spending on prevention	0.019 (0.029)	
Spending on treatment		0.105 (0.060)
Spending on health systems strengthening	0.002 (0.029)	− 0.115 (0.045)*
ln(GDP per capita)	− 0.012 (0.023)	− 0.023 (0.038)
Age dependency ratio	− 0.001 (0.001)	0.003 (0.002)
Healthcare Access and Quality Index	− 0.011 (0.004)*	− 0.000 (0.007)
Average maternal education	0.064 (0.022)**	0.010 (0.037)
Population-weighted mean temperature	0.028 (0.014)*	0.004 (0.022)
National malaria incidence rate	− 0.274 (0.062)***	− 0.289 (0.103)**
Number of observations	440	438
R^2^	0.142	0.127
Adjusted R^2^	0.009	− 0.009
F statistic	6.291 (df = 10; 380)***	5.491 (df = 10; 378)***

Results based on a fixed effects model for the Gini coefficient

Number of clusters = 40

***Significant at the 0.001 level

**Significant at the 0.01 level

*Significant at the 0.05 level

A 10% increase in total malaria spending per person at risk was associated with 0.002 (95% confidence interval: 0.002–0.007) and 0.003 (0.002–0.011) unit decrease in incidence inequality and case fatality inequality, respectively. Spending on health systems strengthening was associated with a decrease of 11.5% (2.7–20.3) in case fatality inequality. Additionally, national malaria incidence rate was associated with decreases in both incidence and case fatality inequality. In perspective, every increase of 1,000 per 100,000 in incidence is associated with 0.274% (0.152–0.397) and 0.289% (0.087–0.491) decreases in incidence and case fatality inequality, respectively. A one-unit change in the healthcare access and quality index was associated with a 1.1% (0.2–2.0) decrease in incidence inequality. Conversely, government malaria expenditure was associated with an increase of 19.8% (10.4–29.1) in case fatality inequality. A one-degree increase in population-weighted mean temperature was related to a 2.8% (0.2–5.5) increase in incidence inequality. Finally, an additional year of maternal education was associated with a 6.4% (2.0–10.8) increase in incidence inequality.

We further stratified countries by burden of malaria to test for a possible differential effect of malaria spending on outcome inequality. The national incidence rate in 2020 ranged from 0.05 in Senegal to 0.38 in Benin. Countries in the top 50th percentile for burden of malaria included the Solomon Islands and 19 countries in sub-Saharan Africa with an incidence rate greater than 0.228 in 2020. Among high-burden countries, this sensitivity analysis indicated that higher total malaria spending and average maternal education were associated with reductions in malaria outcome inequality. Additionally, government contribution to malaria spending and national malaria incidence were associated with decreases in incidence inequality. Inversely, proportionally higher spending on treatment, GDP per capita, and the age dependency ratio were associated with increases in case fatality inequality. Further, broader development (i.e. GDP per capita and working age population) and environmental conditions (i.e. population-weighted mean temperature) are associated with increases in case fatality and incidence inequality, respectively.

Two additional sensitivity analyses were performed. The first replicated the main analysis using country fixed effects, as opposed to country and time fixed effects, as an alternative model parameterization to assess the potential impact of time. The second used the Gini coefficient for mortality as an alternative outcome measure to case fatality inequality. Although case fatality may be a stronger and more focused indication of the efficacy of and inequalities in spending on treatment, mortality rates may be a stronger indicator of the burden of malaria within the entire population. Both analyses produced similar results to the primary analyses. However, a greater proportion of total spending that is sourced from governments was associated with a decrease in mortality inequality. The results of these sensitivity analyses are presented in Appendix Section C.

## Discussion

The study investigated country-level drivers and characteristics associated with a measure of inequality for two malaria outcomes across 40 malaria-endemic countries. Total and government malaria spending, spending on health systems strengthening for malaria, maternal education, and population-weighted mean temperature were identified as important drivers of inequality in malaria outcomes within countries. The findings from the study showed how malaria elimination efforts can benefit from quantifying the variation in these drivers of inequality across population groups and geographic areas. A more precise understanding of the patterns of malaria burden can inform government decision-making, while also helping external funders allocate resources to those areas with the greatest need.

This study is the first to demonstrate how malaria spending through different mechanisms can impact the inequality of disease incidence and case fatality within countries; previous studies focused solely on quantifying geographic variation within individual countries, or variation in malaria-related inequality at the regional level [15]. This study is also the first to apply the Gini index to all countries where malaria continues to cause significant health burden over time. The practical advantage of using the Gini index is that it is a widely used, simple, and standardized measure that is not sensitive to extreme values in malaria outcomes. As such, use of the Gini index in this context provides a comprehensive assessment from which individual countries can assess their performance in malaria elimination against other countries with similar endemicity.

The results from the study highlight some inequality in malaria burden, with average values of the Gini coefficient being 0.20 and 0.18 for incidence inequality and case fatality inequality, respectively, in 2020. While inequality has declined between 2010 and 2020, there is still remains a need to prioritize reducing the malaria burden equitably within countries. The results are in line with previous studies of inequality in malaria outcomes, but the values for within-country malaria inequality in this study are generally lower than those shown in other studies (Fig. 2). For example, a cross-sectional analysis of malaria incidence inequality within Sierra Leone, Nigeria, Ghana, and Burkina Faso found Gini coefficient values ranging from 0.17 to 0.30 between 2015 and 2017 [15], whereas our study had a range of 0.11 to 0.21 for the same countries. Studies using more granular epidemiological surveillance data at the district level also found comparable values for the Gini coefficient for malaria incidence [14, 16].

Malaria morbidity and mortality are strongly influenced by the performance of health systems. The findings suggest an association between a higher proportion of malaria spending on health systems strengthening and improved equity in case fatality. Additionally, the study found that a higher proportion of spending on malaria treatment increased inequities in case fatality in high-burden countries; this may be an indication of differential access to malaria treatment between states. The success of malaria control and elimination programmes is acknowledged to be handicapped by the ability of health systems to deliver high-quality interventions that reach the full population-at-risk [40–42]. Further, work by Sahu et al*.* found health systems strength to be predictive of reductions in malaria burden [43], particularly in high-burden countries. These countries may see the greatest benefit from systems strengthening [44] and the integration of a people-centred approach [42].

Additionally, access to high-quality healthcare has been recognized as both key to improving healthcare delivery and as a necessary driver of universal health coverage. The study found an association between access to quality healthcare (measured using the HAQ Index) and incidence inequality in the main analysis. This finding is aligned with work by O’Meara et al*.* that reinforced the importance of primary care—and the proximity to primary care—to malaria outcomes [45], as well as other work showing lower malaria mortality rates in more prepared health centres in Burkina Faso [46], Uganda [47], Kenya, Namibia, and Senegal [48].

There are some important limitations of this work. To start, the estimates of domestic malaria spending used in this analysis [31] were generated using country-reported data, which is subject to data gaps such as missing data and incomplete documentation and, therefore, required modelled estimation. Interpreting malaria spending estimates is also complicated. For example, while high spending can be attributed to a heavy malaria burden, it may also be influenced by wealthier nations with less burden and their efforts towards eliminating malaria entirely. As such, national spending on malaria treatment, prevention, and health systems strengthening as a proportion of total malaria spending were transformed.

Additionally, the study does not quantify inequality among countries with low national incidence or incidence concentrated in specific areas of countries. This is because Gini coefficients could be skewed by small number bias (i.e. few admin1 units with malaria burden), systematically biasing estimates towards inflated Gini coefficients [49]. As such, the analysis was restricted to relatively high-burden countries only (Tables 1 and 4). While the Gini coefficient is a useful quantitative descriptor of malaria inequality, it is important to note that these values do not represent absolute differences in malaria outcomes and that different distributions of malaria outcomes within countries can produce the same Gini coefficient. Future applications of the Gini coefficient as a standardized measure of disease inequality could consider its use alongside detailed qualitative data exploring determinants of underlying geographic heterogeneity.

**Table 4:** Regression results from a stratified analysis including only countries in the top 50th percentile for national malaria incidence (incidence rate > 22,800 cases per 100,000 in 2020)

	Incidence inequality	Case fatality inequality
ln(total malaria spending)	− 0.034 (0.008)***	− 0.030 (0.010)**
Government malaria expenditure	− 0.070 (0.027)**	0.053 (0.033)
Spending on prevention	0.014 (0.030)	
Spending on treatment		0.165 (0.048)***
Spending on health systems strengthening	− 0.003 (0.026)	0.010 (0.031)
ln(GDP per capita)	− 0.030 (0.022)	0.070 (0.028)*
Age dependency ratio	− 0.002 (0.001)	0.002 (0.001)*
Healthcare Access and Quality Index	− 0.003 (0.004)	0.000 (0.005)
Average maternal education	− 0.053 (0.021)*	− 0.083 (0.027)**
Population-weighted mean temperature	0.037 (0.012)**	0.000 (0.015)
National malaria incidence rate	− 0.312 (0.051)***	− 0.079 (0.066)
Number of observations	220	220
R^2^	0.328	0.330
Adjusted R^2^	0.183	0.184
F statistic	8.802 (df = 10; 180)***	8.851 (df = 10; 180)***

Results based on a fixed effects model for the Gini coefficient

Number of clusters = 20

***Significant at the 0.001 level

**Significant at the 0.01 level

*Significant at the 0.05 level

The series analysis was restricted to 2010 to 2020 as admin1 level data of incident cases and deaths from MAP were only available for this period. This study also employs cross-sectional methodologies for time series analysis, which does not imply causality between the predictors and outcomes. Therefore, it is important to note that these evaluations are all descriptive and not causal. One of the outcomes in the study was defined as ‘spending per person at risk population’ the authors acknowledge that the level of risk people face may vary because of inequities but the scope for this study does not allow for further investigation of that variation. Nevertheless, the findings provide an important foundation for initiating discussions on plausible causal theories in settings characterized by high inequalities in malaria burden.

Inequalities have been widely acknowledged as barriers to achieving global and national targets in malaria programs. To realize global malaria elimination, routine surveillance activities should include inequality monitoring at lower geographic levels from which methods to address existing gaps can be drawn. This paper provides an important policy message with implications for both national governments and the international community. Results from this study highlight the responsiveness of geographic inequalities in malaria incidence and case fatality to the way in which financial resources for malaria are allocated, as well as to broader drivers of economic development. While the finding that more funding improves malaria outcomes is not new, the finding that increased funding may also reduce geographic inequalities is new. This highlights the potential double impact of malaria programme investments. It is, therefore, essential that countries controlling for malaria continue to prioritize efficiency in their resource investments to be able to improve upon their malaria outcomes and geographic inequalities despite limited availability of additional financial resources. Furthermore, even though some of the results suggest that a greater proportion of spending on malaria treatment is associated with greater case fatality inequality, this is not suggesting that treatment should not be prioritized but rather that ensuring equity in treatment availability should be further prioritized.

## Conclusion

The results presented in this paper highlight the large heterogeneity in malaria outcomes between 2010 and 2020 within malaria-endemic countries. The analysis further shows the high impact of allocating greater resources towards health systems strengthening and improving healthcare access and quality in the reduction of inequality of malaria burden within countries. This indicates the potential double impact of stronger health systems and in addressing distributional challenges related to the attainment of elimination and eradication goals across countries.

### Supplementary Information

Supplementary Material 1.

Supplementary Material 2.

## Data Availability

The data used in the analysis is available in the supplemental appendix.

## Abbreviations

GDP: Gross domestic product
HAQ index: Healthcare Access and Quality index
HSS: Health systems strengthening
MAP: Malaria Atlas Project
ITN: Insecticide-treated nets
IRS: Indoor residual spraying
HRTA: Human resources and technical assistance
PSM: Procurement and supply chain management
PAO: Planning, administration, and overheads
IE: Infrastructure and equipment
ME: Monitoring and evaluation
