# Publisher Correction: Examining geographical inequalities for malaria outcomes and spending on malaria in 40 malaria-endemic countries, 2010–2020

**DOI:** 10.1186/s12936-024-05065-z

**Published:** 2024-08-19

**Authors:** Angela E. Apeagyei, Nishali K. Patel, Ian Cogswell, Kevin O’Rourke, Golsum Tsakalos, Joseph Dieleman

**Affiliations:** Institute for Health Metrics and Evaluation, 3980 15th Ave NE, Seattle, WA 98195 USA

**Publisher Correction: Malaria Journal (2024) 23:206** 10.1186/s12936-024-05028-4

Following publication of the article [1], it came to the publisher’s attention that the joint first authors had not been correctly indicated in the author list: the dagger ‘symbol’ used to denote shared first authorship was missing. This symbol has since been added to the authorship list of the article. The publisher thanks you for reading and apologizes for any inconvenience caused.
