# Supplementary material for: Examining geographical inequalities for malaria outcomes and spending on malaria in 40 malaria-endemic countries, 2010–2020
Source: Malar J. 2024 Jul 10;23:206. doi: 10.1186/s12936-024-05028-4 (PMC11234708; doi:10.1186/s12936-024-05028-4)
Supplement: Supplementary file 1 — Supplementary Material 1. [file 12936_2024_5028_MOESM1_ESM.docx]

Supplementary Appendix

Title: Examining geographical inequalities for malaria outcomes and spending on malaria in 43 malaria-control countries, 2010–2020

Authors: Angela E. Apeagyei*, Nishali K. Patel*, Ian Cogswell, Kevin O’Rourke, Golsum Tsakalos, Joseph Dieleman

* Joint first authors

Affiliations: 1 Institute for Health Metrics and Evaluation, University of Washington, Seattle, WA, USA

       2 Department of Health Metrics Sciences, University of Washington, School of Medicine, Seattle, WA, USA

Corresponding: Angela E. Apeagyei (amicah@uw.edu)

List of Appendix Contents:

Appendix A: WHO Conceptual Framework on the Determinants of Health Equity

Appendix B: Lorenz curves for malaria incidence, mortality, and case fatality inequality

Appendix C: Regression results

Appendix A

WHO’s conceptual framework on the determinants of health equity


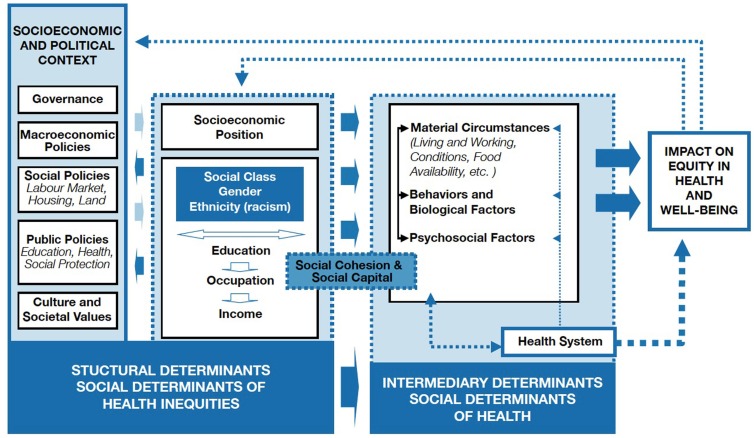


**Source:** Solar O, Irwin A. A conceptual framework for action on the social determinants of health. Social Determinants of Health Discussion Paper 2 (Policy and Practice)

Appendix B

**Fig B1. The Lorenz curves of the distribution of malaria incidence, 2010 - 2020**


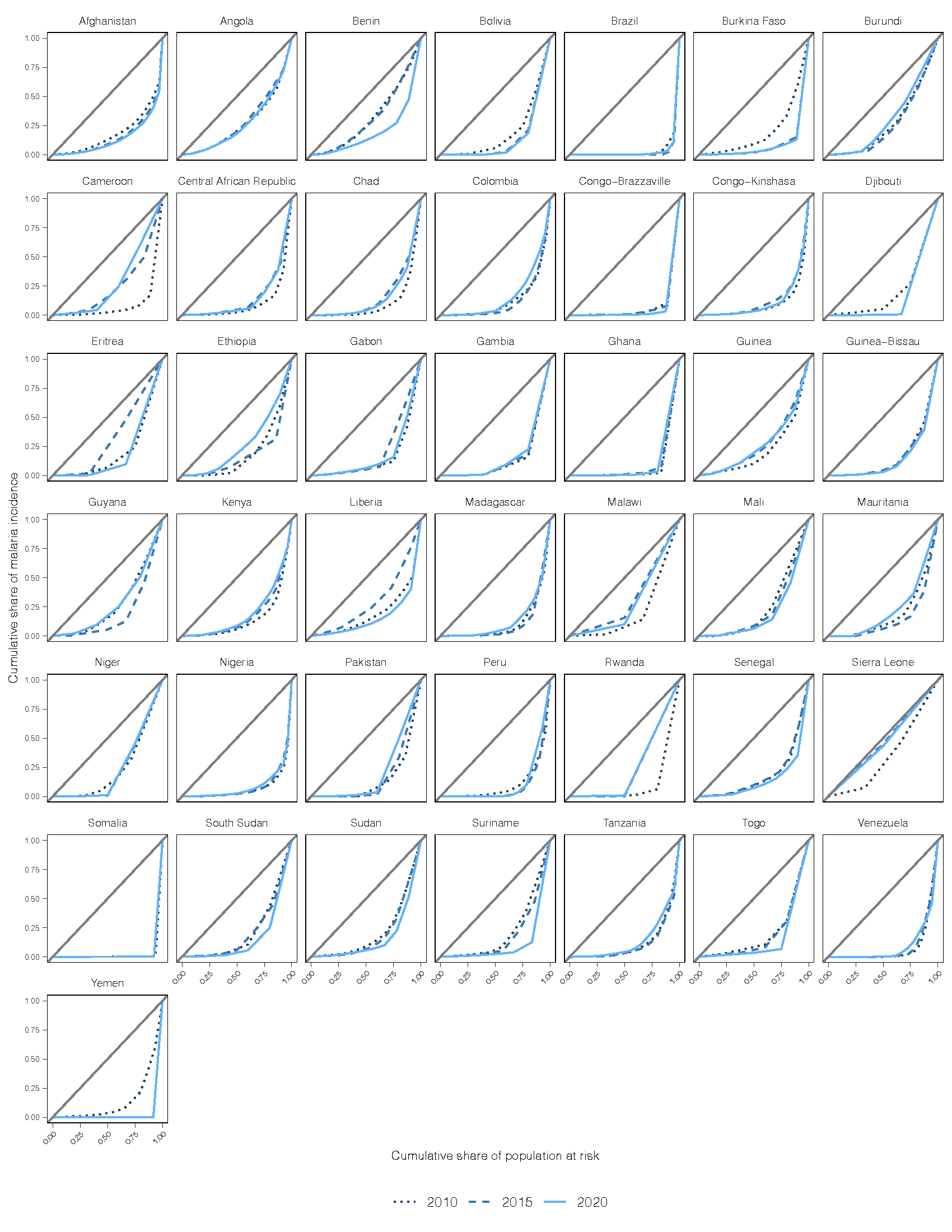


**Fig B2. The Lorenz curves of the distribution of malaria case fatality, 2010 - 2020**

**
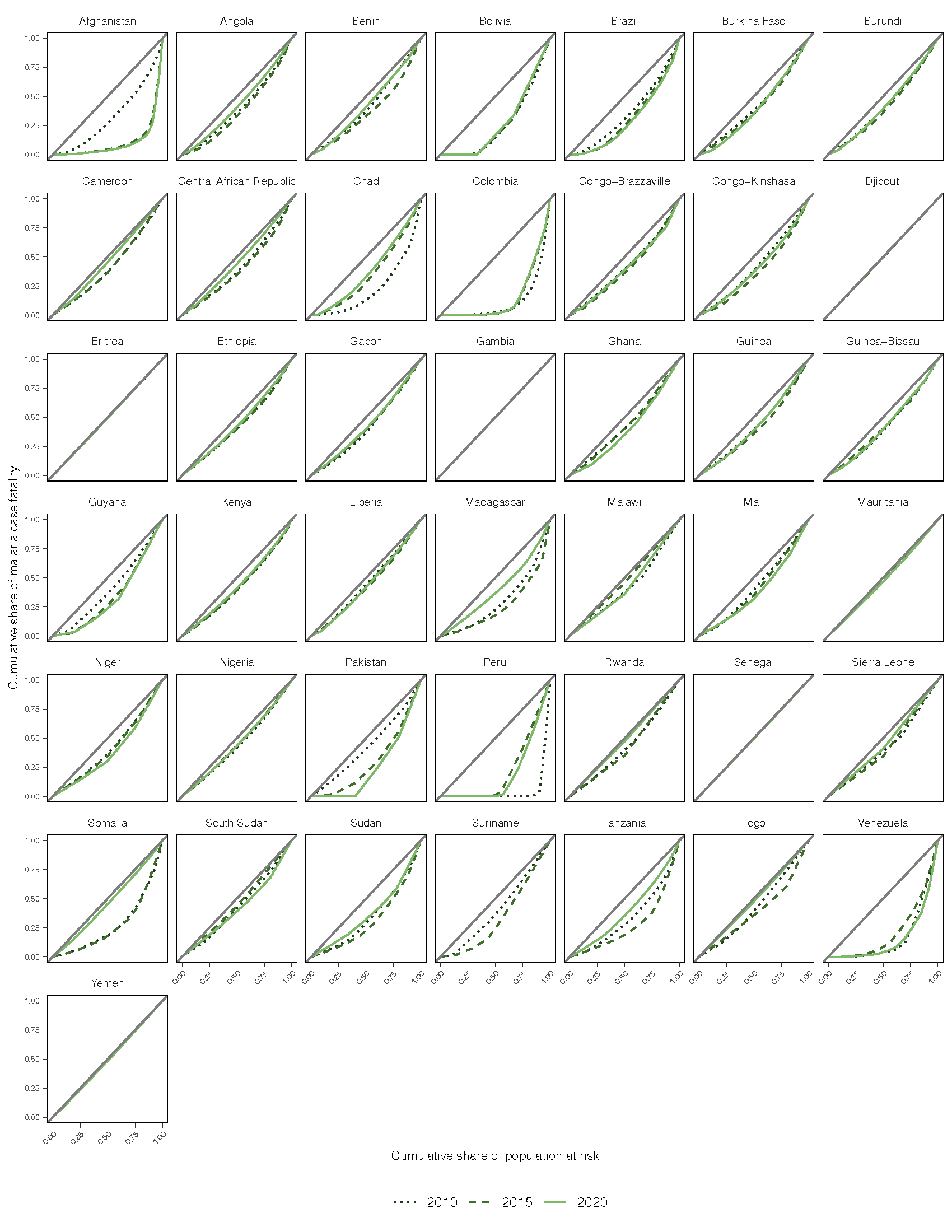
**

Appendix C

**Table C1: Regression results using country only fixed effects (admin1 state-level outcomes)**

|  | **Incidence** | **Case fatality** | |
| --- | --- | --- | --- |
| **ln(Total malaria spending)** | -0.027 (0.013) | 0.014 (0.015) | |
| **Gov’t malaria spending** | 0.084 (0.066) | 0.075 (0.065) | |
| **Spending on treatment** | - | -0.004 (0.083) | |
| **Spending prevention** | -0.058 (0.055) | - | |
| **Spending on health systems strengthening** | -0.138 (0.044) ^***^ | -0.144 (0.052) ^***^ | |
| **National malaria incidence** | -0.092 (0.095) | -0.014 (0.087) | |
| **Healthcare access and quality index** | -0.004 (0.005) | -0.011 (0.006) ^*^ | |
| **Age-dependency ratio** | -0.001 (0.002) | -0.002 (0.002) | |
| **ln(GDP per capita)** | -0.077 (0.037) ^**^ | 0.041 (0.036) | |
| **Average maternal education** | 0.015 (0.020) | 0.026 (0.025) | |
| **Population-weighted mean temperature** | 0.025 (0.013) ^*^ | 0.019 (0.016) | |
| ***Number of observations*** | 471 | 468 | |
| ***R^2^*** | 0.055 | 0.041 | |
| ***Adjusted R^2^*** | -0.063 | -0.079 | |
| ***F statistic*** | 2.435 (df = 10; 418)^***^ | 1.761 (df = 10; 415) ^*^ | |
| Results based on a fixed effects model for the Gini coefficient  Number of clusters = 43  *** significant at the 0.01 level, ** significant at the 0.05 level, * significant at 0.10 level | | |  |

**Table C2: Regression results using mortality inequality as the outcome (admin1 state-level outcomes)**

|  | **Model 1 (country and time fixed effects)** | **Model 2 (country only fixed only)** | | | **Model 3 (Stratified analysis)** | |
| --- | --- | --- | --- | --- | --- | --- |
| **ln(Total malaria spending)** | 0.007 (0.014) | 0.005 (0.013) | -0.005 (0.028) | | | |
| **Gov’t malaria spending** | 0.075 (0.065) | 0.080 (0.066) | -0.096 (0.110) | | | |
| **Spending on treatment** | 0.136 (0.071) ^*^ | 0.168 (0.069) ^**^ | 0.067 (0.229) | | | |
| **Spending prevention** | -0.059 (0.064) | -0.034 (0.062) | -0.085 (0.123) | | | |
| **Spending on health systems strengthening** | -0.114 (0.049) ^**^ | -0.091 (0.046) ^**^ | -0.063 (0.101) | | | |
| **National malaria incidence** | -0.091 (0.096) | -0.135 (0.105) | -0.321 (0.121) ^**^ | | | |
| **Healthcare access and quality index** | -0.009 (0.006) | -0.001 (0.006) | 0.001 (0.012) | | | |
| **Age-dependency ratio** | -0.002 (0.002) | -0.002 (0.002) | 0.004 (0.002) | | | |
| **ln(GDP per capita)** | -0.102 (0.038) ^***^ | -0.113 (0.038) ^***^ | -0.018 (0.056) | | | |
| **Average maternal education** | -0.098 (0.026) ^***^ | 0.009 (0.022) | -0.168 (0.051) ^**^ | | | |
| **Population-weighted mean temperature** | 0.024 (0.017) | 0.014 (0.014) | 0.026 (0.032) | | | |
| ***Number of observations*** | 468 | 468 | 230 | | | |
| ***R^2^*** | 0.075 | 0.052 | 0.096 | | | |
| ***Adjusted R^2^*** | -0.069 | -0.070 | -0.102 | | | |
| ***F statistic*** | 2.980 (df = 11;404) ^***^ | 2.053 (df = 11; 414) ^**^ | 1.800 (df = 11;188) ^*^ | | | |
| Results based on a fixed effects model for the Gini coefficient  Number of clusters for Models 1 and 2 = 43  Number of clusters for Model 3 = 20  *** significant at the 0.01 level, ** Significant at the 0.05 level, * significant at 0.10 level | | | |  | |  |
